# Assessment of disease specific immune responses in enteric diseases using dried blood spot (DBS)

**DOI:** 10.1371/journal.pone.0218353

**Published:** 2019-06-17

**Authors:** Md Saruar Bhuiyan, Motaher Hossain, Salma Sharmin, Afsana Shirin, Farhana Khanam, Fahima Chowdhury, Afroza Akter, Ashraful Islam Khan, Muhammad Ikhtear Uddin, Taufiqur Rahman Bhuiyan, Firdausi Qadri

**Affiliations:** icddr,b (International Centre for Diarrhoeal Disease Research, Bangladesh), Dhaka, Bangladesh; George Washington University School of Medicine and Health Sciences, UNITED STATES

## Abstract

**Background:**

Blood collection, transportation and storage remain a problem in countries where infrastructure, laboratory facilities and skilled manpower are scarce. This limits evaluation of immune responses in natural infections and vaccination in field studies. We developed methods to measure antigen specific antibody responses using dried blood spot (DBS) in cholera, ETEC and typhoid fever patients as well as recipients of oral cholera vaccine (OCV).

**Methodology/Principle findings:**

We processed heparinized blood for preparing DBS and plasma specimens from patients with, cholera, ETEC and typhoid as well as OCV recipients. We optimized the conventional vibriocidal method to measure vibriocidal antibody response in DBS eluates. We measured responses in DBS samples and plasma (range of titer of 5 to 10240). Vibriocidal titer showed strong agreement between DBS eluates and plasma in cholera patients (ICC = 0.9) and in OCV recipients (ICC = 0.8) using the Bland-Altman analysis and a positive correlation was seen (r = 0.7, p = 0.02 and r = 0.6, p = 0.006, respectively). We observed a strong agreement of lipopolysaccharide (LPS) and cholera toxin B (CTB)-specific antibody responses between DBS eluates and plasma in cholera patients and OCV recipients. We also found agreement of heat labile toxin B (LTB) and membrane protein (MP)-specific antibody responses in DBS eluates and plasma specimen of ETEC and typhoid patients respectively.

**Conclusion:**

Our results demonstrate that dried blood specimens can be used as an alternate method for preservation of samples to measure antibody responses in enteric diseases and vaccine trials and can be applied to assessment of responses in humanitarian crisis and other adverse field settings.

## Introduction

Blood collection, processing and quick transportation to laboratories from remote areas in resource poor settings can be limitations in carrying out serological studies especially in developing countries where laboratory facilities and trained manpower as well as cold chain facilities are not optimal. Dried blood spot (DBS) is an alternate method for collecting blood specimens on paper. This lowers biological risks associated with conventional blood transportation, does not require maintaining cold-chain during transportation and DBS card can be shipped at ambient temperature and even by ordinary mail [1,2]. Blood collection and transportation from resource-limited settings remains an obstacle to follow up large vaccine trials, seroepidemiological studies and diagnosis of infectious diseases as well as large population based estimations of public health interventions. DBS can therefore be a solution to the cumbersome specimen collection and transportation that is generally used.

DBS has been used as an alternative method for diagnosis of viral [3–5], bacterial [6], fungal [7] and parasitic [6] infections. It has also been used to monitor HIV viral load and drug resistance in patients receiving antiretroviral therapy [1]. The DBS analysis platform is routinely used for estimation of DNA, protein and drugs [8–11] which have a great impact in clinical practice. DBS has been used for measuring immune responses after hepatitis A virus vaccination [12] with a positive agreement with plasma specimens. However, there is lack of information about the sensitivity of DBS to evaluate the immunogenicity in enteric infections.

In this study, we have used the DBS method to collect blood from patients with cholera, enterotoxigenic *Escherichia coli* (ETEC) diarrhea, typhoid fever as well as recipients of an oral cholera vaccine to evaluate feasibility and accuracy of immune responses. Immune responses measured using the DBS method was compared with results obtained with plasma specimens in the same cohort of patients and vaccinees. There was positive agreement between DBS and plasma specimen in the evaluation of immune responses in cholera, ETEC diarrhea and typhoid patients as well as OCV recipients.

## Methods

### Study participants

Blood specimens were obtained from patients with enteric infections which included cholera, ETEC, typhoid and as well as healthy participants who received two doses of heat-killed oral cholera vaccine (Shanchol, Shantha biotechnics, India). Cholera and ETEC-infected patients were culture confirmed by isolation of *Vibrio cholerae* O1 and enterotoxigenic *Escherichia coli* (ETEC) from stool specimens respectively. ETEC was confirmed by the presence of heat-labile toxin (LT) or heat-stable toxin (ST) or both by ELISA and PCR [13]. Typhoid fever was confirmed by isolation of *Salmonella* Typhi from blood of febrile patients. Patients were given appropriate antibiotics by physicians as was needed (S1 Table). Specimens were collected between January 2016 and December 2017.

### Ethics statement

Specimen collection from cholera, ETEC and typhoid patients and healthy participants and were approved by the Institutional Research Board (IRB) of icddr,b. Written informed consent was obtained from the study participants (18–59 years), and consent from parents of those aged 5–10 years with assents from the minor participants (11–17 years) were obtained.

### Collection of blood samples in DBS cards

Venous blood was collected (8–10 ml from adults and 3–5 ml from children; only 200 μl was used for preparation of DBS) from the study participants in sodium heparin tube (Beckton Dickinson, CA). Blood was collected from cholera and ETEC diarrheal patients on day 2, 7 and 30 of onset of disease. From typhoid fever patients, blood was collected after culture confirmation (day 1), 2–3 days later (day 2) and 18–22 days later (day 3). Healthy participants were vaccinated with oral cholera vaccine at two time points (days 0 and 14). Blood was collected prior to vaccination on day 0 and 14 (prior to second vaccination) and two more blood samples were collected at day 28 and 42 after the first vaccination. Approximately 50 μl of blood was adsorbed on each spot (200 μl for four spots) of DBS card (Whatman 903, GE Healthcare) and air dried at ambient temperature (15–25°C) for two hours by placing on a clean paper towel. The card was then transferred to a black card holder (Advantus 4X6 index card holder, Jacksonville, FL, USA) box and kept overnight at RT for adequate drying. The DBS card was placed in a single, gas-impermeable zipper bag containing desiccant sachets (2 desiccants per bag) to protect the cards from moisture. A humidity indicator card was added to monitor the moisture level in the zipper bag and it was checked monthly. If the indicator turned purple then the desiccant sachets were replaced with new desiccant sachets. The dried blood spotted cards were stored at -80°C until tested (1–6 months).

### DBS elution

A 3.2 mm spot was punched with a DBS puncher (PerkinElmer, USA) from each blood-soaked circle of the DBS card. All punched spots from a single patient were transferred to one eppendorf tube and 0.5% bovine serum albumin (BSA) in phosphate buffered saline (PBS, 10 mM, pH 7.2) was added according to the desired dilution (18 punch in 270 μl; 1:10 for vibriocidal and 1 punch in 300 μl; 1:200 for ELISAs) and kept at RT overnight. The tube was centrifuged (2500 x g for 7 min) and eluate was collected in a fresh eppendorf tube.

### Plasma separation

Plasma specimens were also separated from the same cohort of patients and vaccinees from the heparinized blood. Blood specimens were centrifuged, aliquoted (200 μl in eppondorf tube) and stored at -20°C until tested.

### Vibriocidal assay

**Fig 1:**
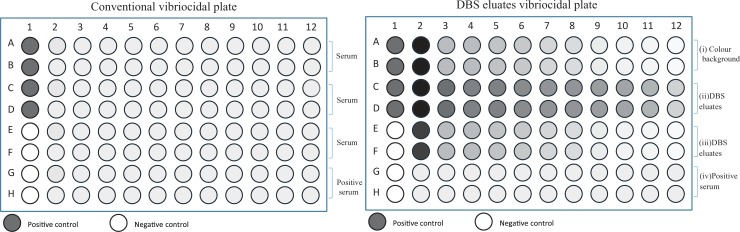
Conventional vibriocidal assay and modified vibriocidal assay for DBS eluates. A) The schematic diagram shows the conventional vibriocidal assay with plasma collected from cholera patients and cholera vaccinees using the 96-well plates (row A-F). Pooled sera obtained from cholera patients was used as positive control on each plate. B). The vibriocidal antibody assay was modified when DBS eluates were used to measure vibriocidal response in cholera patients/vaccinees. The first two rows (rows A-B) were used without adding bacterial inocula to calculate color background of heme in DBS eluates. Rows C to F were used for DBS eluates similar to the conventional vibriocidal assay and the last two rows (G-H) were used as positive control.

Vibriocidal antibody titer was measured according to the procedure described earlier [14]. Briefly, 25 μl of cold physiological saline (0.154 M) was dispensed in all wells except column #2 of 96-well microtiter plates (Nunc F, Denmark). Plasma and DBS eluates were heat inactivated at 56°C for 30 min. For plasma, 45 μl of cold saline and 5 μl of plasma and for DBS eluates 50 μl eluates were dispensed in corresponding wells in column #2. Both plasma and DBS eluates were serially diluted (initial dilution 1:10) in two fold dilutions. The dilution was accomplished by mixing the solution in column #2, aspirating 25 μl and dispensing and mixing the sample in column #3 and so on, until column #12 (1:10240 dilution). At the end, 25 μl was discarded from the last well in each row. *V*. *cholerae* O1 Inaba strain (T-19479) was cultured on blood agar plates at 37°C overnight. This strain was isolated from stool of cholera patient [15] in Bangladesh and well characterized [16–19] as well as used to measure vibriocidal antibody titer in plasma [14]. A loopful of bacteria was inoculated in Brain Heart Infusion (BHI) media for 2–3 hours at 37°C with shaking to mid-log phase of growth (OD to 0.4). Bacterial culture was centrifuged and resuspended in sterile saline (0.154 M) and the OD was adjusted to 0.3 at 600 nm using a spectrophotometer (Beckman DU 530 UV vis). For vibriocidal assay using plasma, 25 μl of bacterial suspension (21.25 μl normal saline, 1.25 μl bacteria and 2.5 μl guinea pig sera) was added to all wells except wells #E1, F1, G1 and H1 in column 1 of 96-well plates and 25 μl of cold saline was added to wells # E1, F1, G1 and H1 (Fig 1A). For vibriocidal assay using DBS, 25 μl of bacterial suspension (21.25 μl normal saline, 1.25 μl bacteria and 2.5 μl guinea pig sera) was added to all wells except wells row #A2-A12, B2-B12 wells and column #E1, F1, G1 & H1 wells and 25 μl of cold saline was added to wells # E1, F1, G1 and H1 (Fig 1B). The OD value of wells in row #A2-A12, B2-B12 was used as the color background control for DBS (Fig 1B). The plates were incubated at 37°C for 1 hour and 150 μl of BHI media was added to all wells and incubated again at 37°C. The plates were read spectrophotometrically (Eon, BioTek, Vermont, USA) until the positive control well reached OD around 0.20 to 0.28 at 595 nm.

### *V*. *cholerae* lipopolysaccharide (LPS) extraction

LPS was purified from *V*. *cholerae* strains as discussed previously [20]. Briefly, LPS was purified by the hot phenol-water procedure, ultracentrifugation and treatment with enzymes (proteinase K, DNase and RNase) from *V*. *cholerae* O1 Inaba (T-19479) strain.

### *V*. *cholerae* LPS and CTB specific antibody responses in cholera patients and vaccines

For this assay, 96-well ELISA (Nunc F, Denmark) plates were coated with LPS of *V*. *cholerae* O1 in PBS (100 μl/well) at a concentration of 2.5 μg/ml and incubated at RT overnight. For anti-CTB, ELISA plates were coated (100 μl/well) with ganglioside GM1 (0.3 nM/ml) and incubated overnight at ambient temperature (15–25°C). The plates were washed with PBS and recombinant CTB (0.5 μg/ml) (gift of A.M Svennerholm, University of Gothenburg) added to the wells and incubated for 1 h at 37°C. The plates were washed for three times with PBS and blocked with 200 μl/well of 0.1% BSA-PBS and incubated at 37°C for 30–60 min. The plates were washed three times with PBS-0.05% Tween and once with PBS. Plasma and DBS eluates were added in the allocated wells (1:200 dilution) and incubated for 90 min at 37°C. The plates were washed three times with PBS-0.05% Tween and once with PBS. Horseradish peroxidase (HRP) conjugated anti-human antibody was added in each well (100 μl/well) and incubated for 90 min at 37°C. The plates were washed three times with PBS-0.05% Tween and once with PBS. For color development, orthophenylenediamine (OPD) (Sigma, St. Louis, MO) was added together with the substrate H_2_O_2_ (100 μl/well) [10 mg OPD in 10 mL 0.1M Citrate Buffer (pH-4.5), 4 μl 30% H_2_O_2_] to each well and OD measured at 450 nm using the kinetic method (Eon, BioTek, Vermont, USA).

### Membrane protein antigen of *S*. Typhi specific antibody responses in typhoid fever patients

MP was prepared from *S*. Typhi by using a previously described procedure [21, 22]. Briefly, *S*. Typhi strain (Ty21a) was cultured on sheep blood agar plates and harvested in Tris buffer (10 mM Tris [pH 8.0], 5 mM MgCl_2_). Mixture was sonicated and centrifuged at 1400 x g for 10 min, supernatant transferred to fresh tubes, centrifuged again at 14900 x g for 30 min. The pellet was suspended in 10 ml of Tris buffer, and the protein content was determind [22]. Antibody responses in plasma as well as DBS of typhoid fever patients against MP [22, 23] were measured as described previously [22]. Briefly, 96-well ELISA (Nunc F, Denmark) plates were coated with MP of *S*.Typhi (Ty21a) in PBS at a concentration of 2.5 μg/ml and incubated at RT overnight. The plates were washed for three times with PBS and blocked with 200 μl/well of 0.1% BSA-PBS and incubated at 37°C for 30–60 min. The plates were washed three times with PBS-0.05% Tween and once with PBS. Plasma and DBS eluates were added in the allocated wells and incubated for 90 min at 37°C. The plates were washed three times with PBS-0.05% Tween and once with PBS. Horseradish peroxidase conjugated anti-human antibody was added in each well (100 μl/well) and incubated for 90 min at 37°C. The plates were washed three times with PBS-0.05% Tween and once with PBS. Orthophenylenediamine (OPD) with H_2_O_2_ (100 μl/well) [10 mg OPD in 10 mL 0.1M Citrate Buffer (pH-4.5), 4 μl 30% H_2_O_2_] was added to each well and OD was measured at 450 nm using the kinetic method [22].

### Antibody responses against LTB in ETEC-infected patients

Plasma antibodies specific to LTB were measured using a previously described ELISA method [24]. Briefly, 96-well ELISA (Nunc F, Denmark) plates were coated (100 μl/well) with ganglioside GM1 (0.3 nM/ml) and incubated overnight at ambient temperature (15–25°C). The plates were washed with PBS and recombinant LTB (0.5 μg/ml) (gift of A.M Svennerholm, University of Gothenburg) added to the wells (100 μl/well) and incubated for 1 h at 37°C. The plates were washed with PBS-T (0.05%) for three times and once with PBS. The plasma samples (1:200 dilution) was added and incubated for 90 min at 37°C. The plates were washed three times with PBS-T(0.05%) and once with PBS. HRP-conjugated anti-human antibody was added to wells and incubated for 90 min at 37°C. The plates were washed three times with PBS-T (0.05%) and once with PBS. Substrate was added to the wells and plates were read at 450 nm using the kinetic method (Eon, BioTek, Vermont, USA).

### Statistical analyses

We assessed differences in the magnitude of the responses using the Mann-Whitney U test using GraphPad prism 6 (GraphPad Software, Inc., La Jolla, CA, USA). Two-sided P values <0.05 were considered significant. Correlations were assessed using Spearman’s rank correlation. The Bland-Altman plots with 95% limits of agreement (LoA) were used to evaluate the agreement between results obtained with plasma and DBS. The 95% LoA was calculated as the average difference between the plasma and DBS ± 1.96SD (upper limit and lower limit) in SPSS Statistics version 20.0 (IBM Corp., Armonk, NY, USA). The narrower the 95% LoA, the better is the agreement. In addition, the reliability of the DBS method was tested by intraclass correlation coefficient (ICC); if the ICCs were closer to 1, the reliability was higher. Figures were generated in GraphPad prism 6.

## Results

### Study participants

The patients and vaccines were followed up to day 30 and day 42 respectively after enrollment and were included in the analysis. Demographic features of the patients and vaccinees are provided in Table 1.

**Table 1:** Demographic features of patients and participants who were enrolled in this study.

	Age yrs (mean±SD)	Number (Female, Male)
**Cholera patients**	**26.5±10.1**	**9, 8**
**Cholera vaccines**	**20.2±13.3**	**8, 9**
**ETEC infected patients**	**23.1±13.4**	**4, 5**
**Typhoid fever patients**	**12.4±9.2**	**7, 5**

### Optimization of vibriocidal assay for DBS eluates

We set up the vibriocidal assay procedure using DBS eluates so that the assay could be measured in an ELISA Reader (Eon, BioTek, Vermont, USA) similar to the way the conventional and existing vibriocidal assay was being used (Fig 1). The heme color was the main obstacle in setting up the vibriocidal assay to directly measure the antibody titer. To alleviate this problem, the color effect seen in the DBS specimens from each participant were used as DBS control to which no bacterial inoculum was added and this value was subtracted from the OD value of DBS vibriocidal row using an average value for negative controls. We defined the DBS blank as serial dilution (1:2) of DBS eluates and added physiological saline (0.154 M) instead of bacterial inoculum as described above. The optical density from the DBS blank was considered for the heme color and was deducted from DBS plus indicator wells. We defined the final OD of DBS plus indicator wells according the following formula:

OD value of DBS plus indicator well = (OD value of DBS plus indicator—OD value of DBS blank) + Average value of negative controls

If this final OD value was less than or equal to half of the average OD value of positive controls then it was considered as 50% killing of the *V*. *cholerae* O1 strain.

### Vibriocidal responses in cholera patients and vaccines

**Fig 2:**
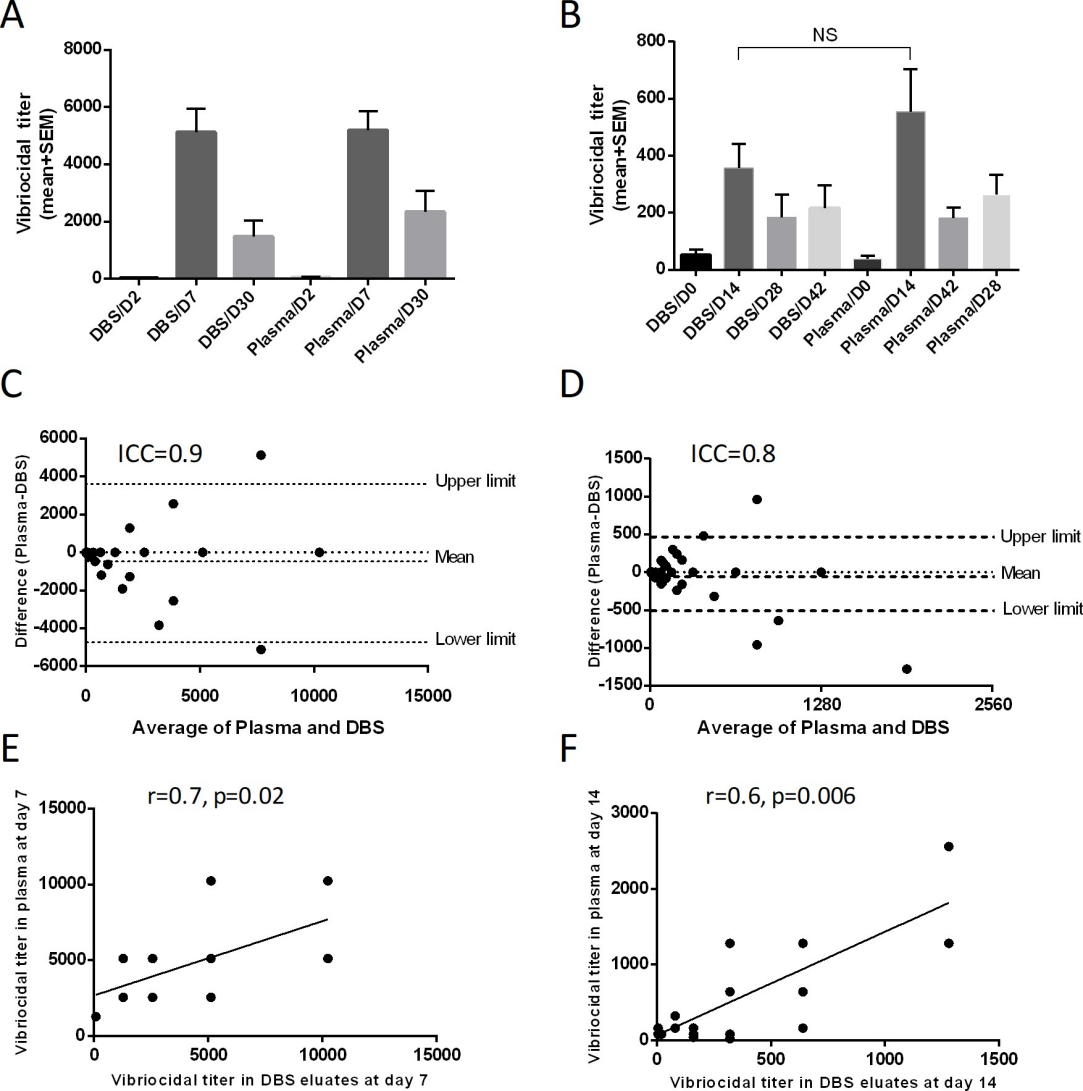
DBS eluates and plasma vibriocidal responses in cholera patients and vaccinees. Vibriocidal responses in cholera patients (A) and vaccinees (B) at different time points (n = 17). The Bland-Altman analysis of vibriocidal response in DBS eluates and plasma of cholera patients at day 7 (C) and vaccinees at day 14 (D) is shown. Spearman correlation of vibriocidal response in DBS eluates and plasma at day 7 of cholera patients (E) and day 14 of vaccinees was carried out (F).

We analyzed the vibriocidal responses in DBS eluates, plasma of cholera patients (n = 17) and vaccinees (n = 17). We found similar magnitude of vibriocidal antibody titer in the corresponding day points of DBS and plasma of patients and vaccinees (Fig 2A and 2B). The responder frequency of vibriocidal titer (four-fold increase) in DBS eluates and plasma (two-fold increase) were similar (90%). There was a strong agreement (Bland-Altman agreement) of vibriocidal responses between DBS eluates and plasma in both patients and vaccinees (ICC = 0.9 and 0.8 respectively; Fig 2C and 2D).There was also a positive correlation of vibriocidal antibody responses at day 7 for patients (r = 0.73, p = 0.02; Fig 2E) and at day 14 for vaccinees (r = 0.6, p = 0.006; Fig 2F). Similar agreement and correlation were observed at other time points in patients and vaccinees (Parts A-D in S1 Fig and Parts A-F in S2 Fig).

### Antibody responses in cholera patients and vaccines

**Fig 3:**
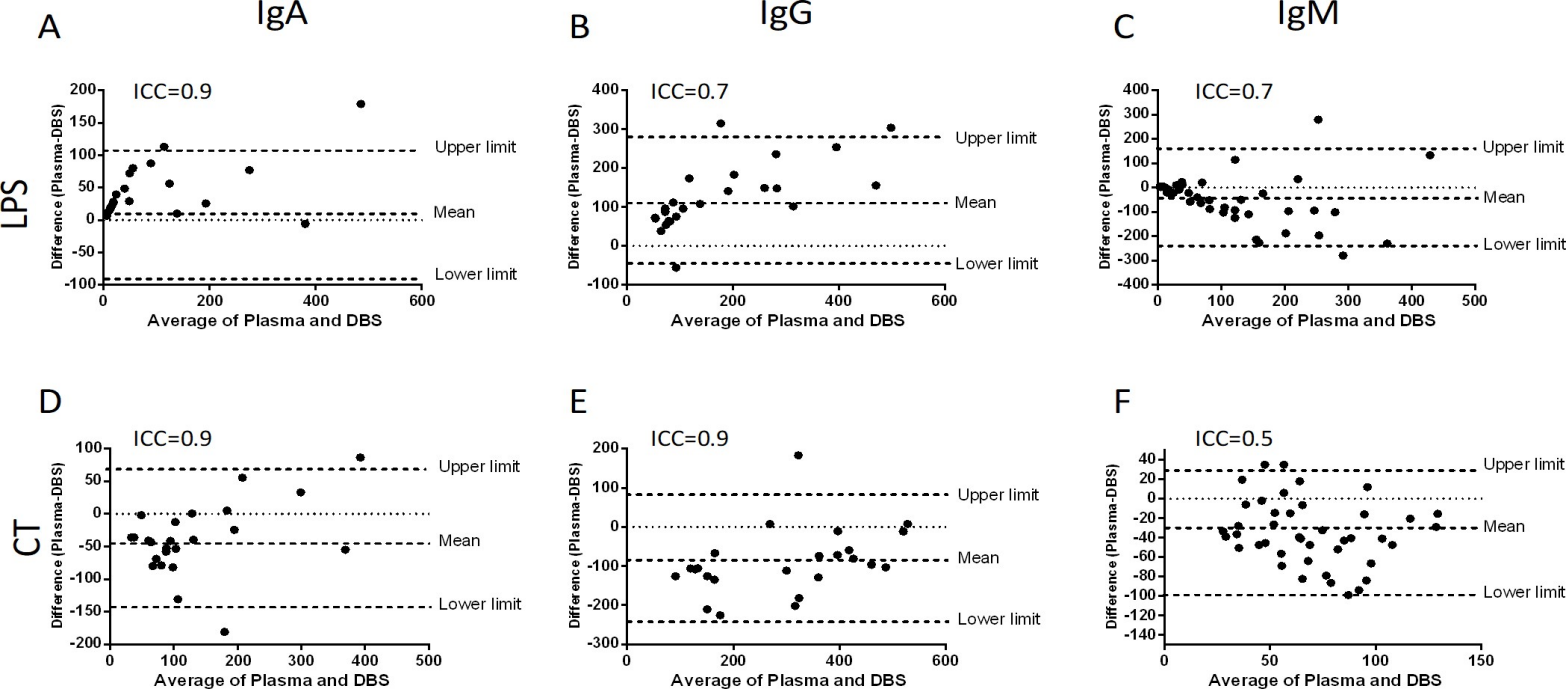
LPS and CTB-specific antibody responses in DBS eluates and plasma in cholera patients. The Bland-Altman analysis is shown for LPS-specific IgA (A), IgG (B), IgM (C) and CTB-specific IgA (D), IgG (E) and IgM (F) antibody responses between DBS eluates and plasma of cholera patients (n = 17) at day 7.

**Fig 4:**
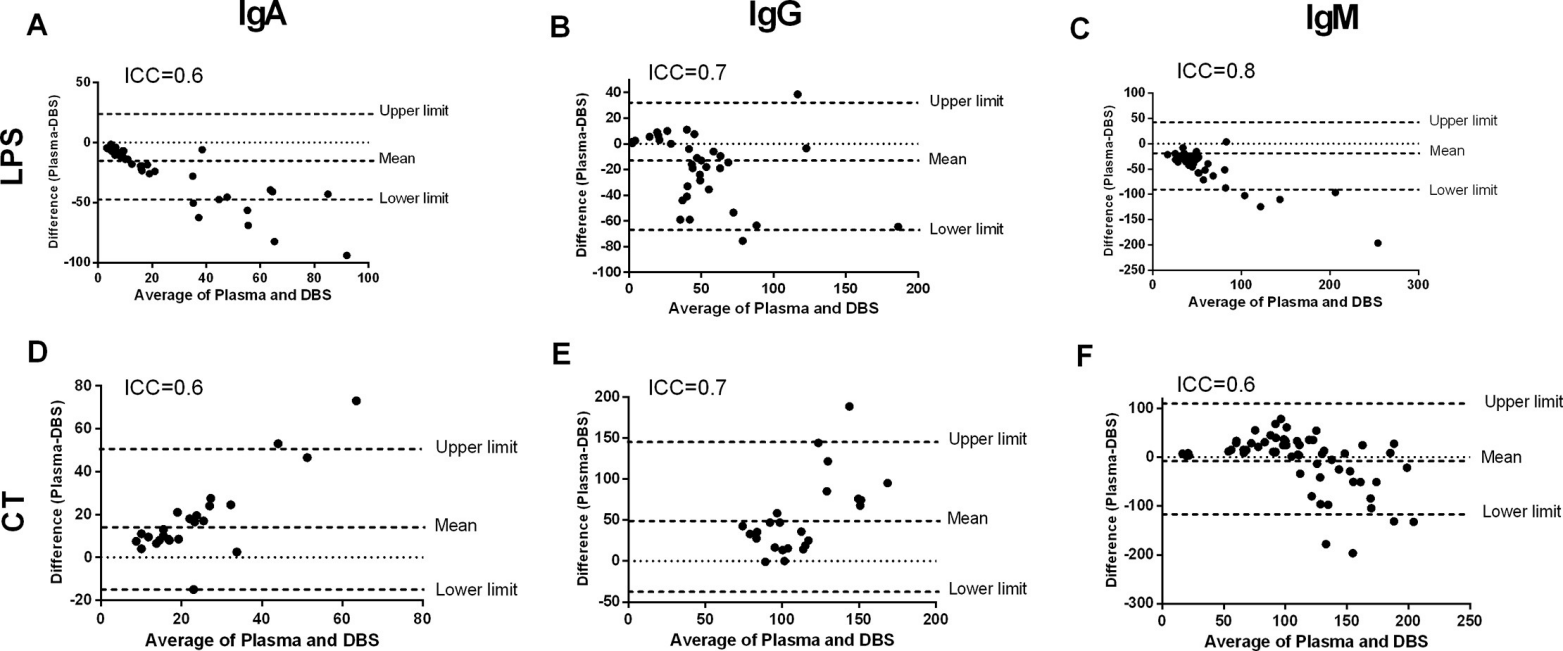
LPS and CTB-specific antibody responses in DBS eluates and plasma in OCV recipients. The Bland-Altman analysis is shown for LPS-specific IgA (A), IgG (B), IgM (C) and CTB-specific IgA (D), IgG (E) and IgM (F) antibody responses between DBS eluates and plasma of OCV recipients (n = 17) at day 14.

We investigated LPS and CTB-specific IgA, IgG and IgM antibody responses in the DBS eluates and plasma specimens of cholera patients and vaccinees. There was a strong agreement of LPS-specific antibody responses between DBS eluates and plasma of cholera patients (Intraclass correlation coefficient, ICC: IgA = 0.9, IgG = 0.7 and IgM = 0.7; Fig 3A–3C) and vaccinees (ICC: IgA = 0.6, IgG = 0.7 and IgM = 0.8; Fig 4A–4C). We also observed a strong agreement of CTB-specific antibody responses between DBS eluates and plasma of cholera patients (ICC: IgA = 0.9, IgG = 0.9 and IgM = 0.5; Fig 3D–3F) and vaccinees (ICC: IgA = 0.6, IgG = 0.7 and IgM = 0.6; Fig 4D–4F). We then investigated the correlation of LPS and CTB-specific antibody responses in DBS eluates and plasma specimens of cholera patients and vaccinees. There was a positive correlation of LPS-specific antibody responses in DBS eluates and plasma specimen of cholera patients (Parts A-C in S3 Fig) and vaccinees (Parts A-C in S4 Fig). Similarly, a positive correlation of CTB-specific antibody responses was observed in DBS eluates and plasma of cholera patients (Parts D-F in S3 Fig) and vaccinees (Parts D-F in S4 Fig).

### Antibody responses in ETEC-infected patients

**Fig 5:**
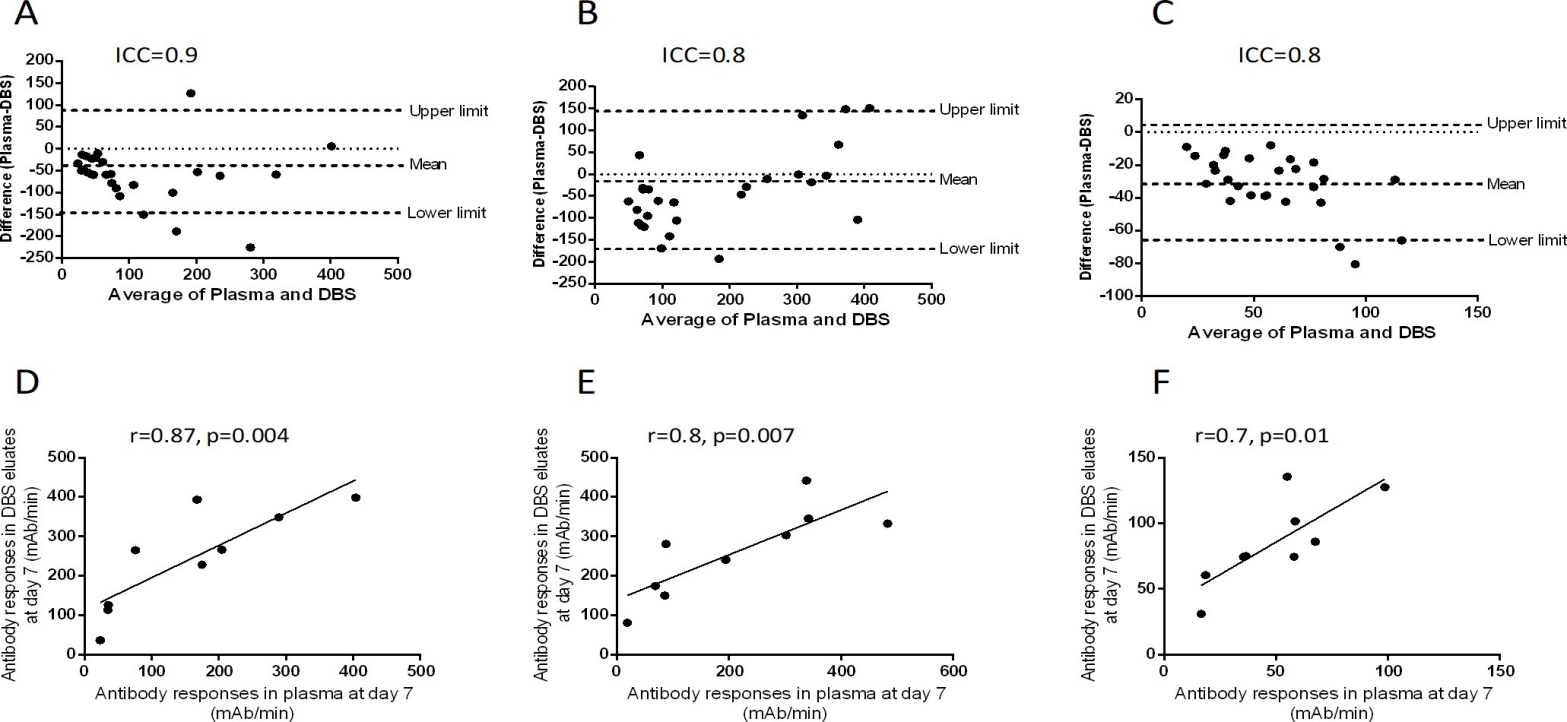
LTB-specific antibody responses in DBS eluates and plasma in ETEC-patients. The Bland-Altman analysis of LTB-specific IgA (A), IgG (B), IgM (C) antibody responses between DBS eluates and plasma specimen of ETEC-patients are shown. Spearman correlation of LTB-specific IgA (D), IgG (E) and IgM (F) between DBS eluates and plasma specimen of ETEC patients.

To investigate the immune responses in ETEC -infected patients we have measured IgA, IgG and IgM responses against LTB antigen by ELISA. There was a strong agreement of LTB-specific antibody responses between DBS eluates and plasma of ETEC-infected patients (ICC: IgA = 0.9, IgG = 0.8 and IgM = 0.8; Fig 5A–5C). There was also a positive correlation of LTB-specific antibody responses between DBS eluates and plasma of ETEC-infected patients at day 7 (IgA: r = 0.87, p = 0.004; IgG: r = 0.8, p = 0.007, and IgM: r = 0.7, p = 0.01). Similar correlations were observed at day 2 and day 30 (S5 Fig).

### Antibody responses in typhoid fever patients

**Fig 6:**
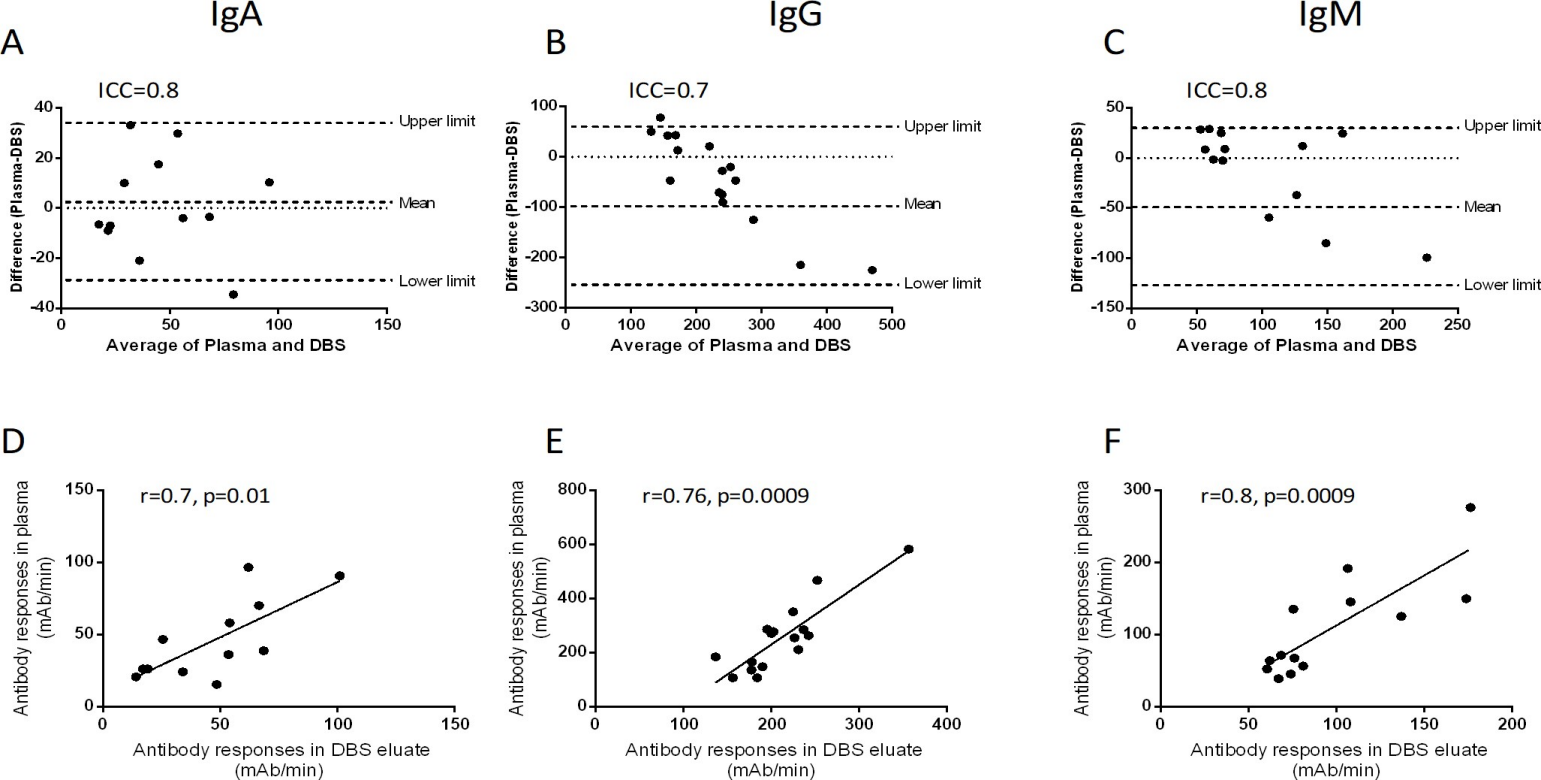
MP-specific antibody responses in DBS eluates and plasma in typhoid fever patients. The Bland-Altman analysis of MP-specific IgA (A), IgG (B), IgM (C) antibody responses between DBS eluates and plasma specimen of typhoid fever patients (n = 12) are shown. Spearman correlation of MP-specific IgA (D), IgG (E) and IgM (F) between DBS eluates and plasma specimen are shown.

We used *S*. Typhi membrane preparation (MP) [21] to measure antibody responses in DBS eluates and plasma specimens of typhoid fever patients. There was a strong agreement of MP-specific antibody responses between DBS eluates and plasma of typhoid fever patients (ICC: IgA = 0.8, IgG = 0.7 and IgM = 0.8; Fig 6A–6C). There was also a positive correlation of MP-specific antibody responses between DBS eluates and plasma of typhoid fever patients at day 1 (IgA: r = 0.7, p = 0.01; IgG: r = 0.76, p = 0.001, and IgM: r = 0.8, p = 0.001; Fig 6D–6F).

## Discussion

Collection and transportation of blood specimen from field settings to laboratories is a problem in resource poor settings and especially in unpredicted epidemics and outbreaks, which often occur in places that have limited laboratory facilities. DBS can also be prepared using finger prick blood since about 50 μl of blood is only required per DBS spot. It is also a less invasive way of blood collection method that this does not necessitate the presence of skilled staff to perform phlebotomy [25]. Thus, the diagnosis and prognosis of diseases and evaluation of disease specific immune responses after natural infection and vaccination can become less difficult to assess and monitor using DBS as an alternative procedure for specimen collection. This procedure as used in this evaluation of enteric diseases and the cholera vaccine brings in an alternative specimen collection and storage method. Although the DBS method has existed for long for sampling for diagnosis of genetic disorders, recently there has been a resurgence of interest in the use of this procedure for carrying out immunological studies in vaccine trials and infectious diseases diagnosis. Enteric infections such as diarrheal diseases (cholera and ETEC diarrhea) as well as typhoid fever are endemic in Africa and Asia. Vaccination is a preventive measure to protect the vulnerable population in these regions. However, to evaluate immunological response to vaccines and natural infection requires collection of blood samples, and transportation to well-equipped laboratories in the cold. We therefore tested the possibility of the use of DBS blood collection method to evaluate immune responses in cholera, ETEC and typhoid patients as well as vaccinees in a country with high rates of enteric infections.

Vibriocidal antibody response is considered one of the key surrogate markers for both natural cholera infection and vaccination in the field of cholera immunology. For the vibriocidal assay, blood needs to be collected to obtain serum or plasma. The procedure requires blood collection and then separation using centrifuges and storage and transportation under cold conditions. All these requires such facilities to be present in remote field settings and peripheral laboratories which are mostly non-existent. In this study, we have demonstrated that the vibriocidal assay can be carried out using dried blood spot, DBS specimens which provide comparable responses to that obtained with plasma in the conventional method. Vibriocidal assay requires measurement of OD values to investigate the growth of *V*. *cholerae* strain. As the DBS eluates are reddish in color because of the presence of heme makes it impossible for measuring vibriocidal antibody in conventional assay. We were able to circumvent the effect of heme in the DBS eluates by subtracting the OD values of the DBS eluates from the color of the background from test wells. This allows us to measure vibriocidal antibody responses in the 96-well plates similar to that used for the conventional vibriocidal assay. This method does not require additional determination of bacterial CFU measurement checking in microbiological culture plates and this saves time, resources and also makes the procedure less costly. The DBS based optimized vibriocidal technique that we developed does not require DBS serum separator card and it is based on a one-step procedure with measurement of optical density of bacterial growth only. However, like the conventional assay it is programmable in 96-well plate ELISA plate reader (Gene 5). Recently Iyer et. al. used DBS serum separator card to separate serum and then performed vibriocidal assay using the drop-plate technique. The drop-plate technique is based on the assumption of determining bactericidal growth or killing using measurement of clumped colonies or serrated margins of growth [26]. However, the method present here does not require additional determination of CFU of bacteria on agar-based culture plate that saves time, resources and money. The method presented here does not require serum separator cards to separate serum for performing the vibriocidal assay [26]. In our opinion, the method described here can replace conventional assay method when only small amount of blood can be obtained from large population and also in field settings with limited resources.

Cholera is water borne disease that can be a cause of death within a few hours if not treated immediately. It has been shown that the oral cholera vaccines can protect people from three to five years from another episode [27–32]. By collecting blood samples from cholera patients and oral cholera vaccinees using the DBS procedure, we have shown that immune responses measured were similar to that seen using plasma. With the DBS usage using finger prick blood collection by venipuncture can be avoided. We also showed that responses to different diseases and antigens could be carried out using DBS specimens in ELISA procedures. In addition, the antibody responses measured in ETEC-infected diarrheal patients to LTB were also comparable when either DBS or plasma was used. Given that we observed a very positive correlation of immune responses in DBS compared to plasma specimen, we extended this observation to typhoid fever patients. The immune responses against membrane preparation (MP) in DBS eluates were also similar to that seen in plasma samples.

DBS has earlier been found to be useful for measuring antibody responses in viral infections e.g. hepatitis C, HIV and Measles [5, 33, 34]. The DBS specimens have also been used for analyzing antibody responses in bacterial, fungal and parasite infections and to our knowledge, there is no published data using DBS for assessing immune response in patients with cholera, ETEC diarrhea and typhoid fever [33–39]. Measuring antibody responses using DBS was found to strongly correlate with plasma antibodies [34, 40, 41]. We show here that the vibriocidal antibody assay which is an indirect correlate of protection in cholera [42] can be measured using the DBS eluates and we hope that this will make it easier to measure immune responses in mass vaccination campaigns as well as in serosurveys for estimating disease burden globally. Likewise large population studies on natural infection or vaccination in areas of typhoid and ETEC diarrhea both natural infection and vaccination as well as other infectious diseases will benefit with the DBS method of sample collection and testing described in this communication.

## Supporting information

S1 Fig Agreement and correlation of vibriocidal responses between DBS eluates and plasma in cholera patients. The Bland-Altman analysis of vibriocidal responses in plasma and DBS eluates of cholera patients at day 2 (A), day 30 (B) are shown. Spearman correlation of vibriocidal responses in plasma and DBS eluates at day 2 (C) and day 30 (D) are shown.
(TIF)

S2 Fig Agreement and correlation of vibriocidal responses between DBS eluates and plasma in OCV recipients. The Bland-Altman analysis of vibriocidal responses in plasma and DBS eluates of OCV recipients at day 0 (A), day 28 (B) and day 42 (C) are shown. Spearman correlation of vibriocidal responses in plasma and DBS eluates at day 0 (D), day 28 (E) and day 42 (F) are shown.
(TIF)

S3 Fig Correlation of LPS and CTB-specific antibody responses in DBS eluates and plasma in cholera patients. Spearman correlation of LPS-specific IgA (A), IgG (B), IgM (C), and CTB-specific IgA (D), IgG (E) and IgM (F) antibody responses between DBS eluates and plasma of cholera patients at day 7 are shown.
(TIF)

S4 Fig Correlation of LPS and CTB-specific antibody responses in DBS eluates and plasma in OCV recipients. Spearman correlation of LPS-specific IgA (A), IgG (B), IgM (C), and CTB-specific IgA (D), IgG (E) and IgM (F) antibody responses between DBS eluates and plasma of cholera patients at day 14 are shown.
(TIF)

S5 Fig Correlation of LTB-specific antibody responses in DBS eluates and plasma in ETEC-patients. Spearman correlation of LTB-specific antibody responses at day 2 [IgA (A), IgG (B), IgM (C)] and day 30 [IgA (D), IgG (E), IgM (F)] between DBS eluates and plasma of ETEC-patients are shown.
(TIF)

S1 Table Organism isolation and antibiotic uses in this study. (DOCX)
