# Supplementary material for: Assessment of disease specific immune responses in enteric diseases using dried blood spot (DBS)
Source: PLoS One. 2019 Jun 17;14(6):e0218353. doi: 10.1371/journal.pone.0218353 (PMC6578496; doi:10.1371/journal.pone.0218353)
Supplement: S1 Table — (DOCX) [file pone.0218353.s006.docx]

**Supporting information**

S1Table: Organism isolation and antibiotic uses in this study.

|  | **Infection** | **Antibiotic used** |
| --- | --- | --- |
| **Cholera patients** | ***V. cholerae* O1** | **Azithromycin** |
| **ETEC infected patients** | **ETEC** | **No antibiotic** |
| **Typhoid fever patients** | ***S.* Typhi** | **Cefixime/Ceftriaxone** |
